# The Transcriptome and Proteome Networks of Malignant Tumours Reveal Atavistic Attractors of Polyploidy-Related Asexual Reproduction

**DOI:** 10.3390/ijms232314930

**Published:** 2022-11-29

**Authors:** Ninel M. Vainshelbaum, Alessandro Giuliani, Kristine Salmina, Dace Pjanova, Jekaterina Erenpreisa

**Affiliations:** 1 Cancer Research Division, Latvian Biomedicine Research and Study Centre, LV-1067 Riga, Latvia; 2 Faculty of Biology, The University of Latvia, LV-1586 Riga, Latvia; 3 Environmen and Health Department, Istituto Superiore di Sanità, 00161 Rome, Italy

**Keywords:** cancer, whole genome duplications, biological interaction networks, gene phylostratigraphy, atavism, basic meiosis attractor, female meiosis, early embryo, pseudo-placentation, soma–germ transition

## Abstract

The expression of gametogenesis-related (GG) genes and proteins, as well as whole genome duplications (WGD), are the hallmarks of cancer related to poor prognosis. Currently, it is not clear if these hallmarks are random processes associated only with genome instability or are programmatically linked. Our goal was to elucidate this via a thorough bioinformatics analysis of 1474 GG genes in the context of WGD. We examined their association in protein–protein interaction and coexpression networks, and their phylostratigraphic profiles from publicly available patient tumour data. The results show that GG genes are upregulated in most WGD-enriched somatic cancers at the transcriptome level and reveal robust GG gene expression at the protein level, as well as the ability to associate into correlation networks and enrich the reproductive modules. GG gene phylostratigraphy displayed in WGD+ cancers an attractor of early eukaryotic origin for DNA recombination and meiosis, and one relative to oocyte maturation and embryogenesis from early multicellular organisms. The upregulation of cancer–testis genes emerging with mammalian placentation was also associated with WGD. In general, the results suggest the role of polyploidy for soma–germ transition accessing latent cancer attractors in the human genome network, which appear as pre-formed along the whole Evolution of Life.

## 1. Introduction

Metastatic cancer is one of the leading causes of death in the world [1,2]. A major determinant of its lethality is the ability of late-stage solid tumours to become increasingly resistant to anti-cancer treatments. Cancer research still lags in understanding the biological reasons for this resistance. Somatic cancers are known to ectopically express the genes related to reproductive processes, such as cancer–testis antigens [3,4,5,6,7] and meiosis-specific genes [7,8,9,10,11,12,13], as well as a wide range of genes specific both to primordial and adult germ cells [14]. Based on the existing overlap between the three aforementioned lists of genes (meiotic, cancer–testis and germ cell-specific), we shall refer to them as a united group of “gametogenesis-related genes” (GG). GG expression is generally associated with a worse patient prognosis [14,15,16,17,18,19]. Reproductive genes paradoxically play a role in promoting the severity and lethality of cancers, which by their origin are somatic. Another notable hallmark of cancer lies in the tendency of tumour cells to acquire whole genome duplications (WGD) and unstable aneuploidy karyotypes, which are also associated with poor outcomes [20]. While at first glance it seems that aneupolyploidy would interfere with mitotic division and proliferation of tumours, it paradoxically favours resistance to treatment, cancer relapse and metastatic growth [21,22,23,24]. This puzzle of cancer could be overcome by the assumption that reproduction-related genes in cancer cells are involved in an atavistic life-cycle-like process ensuring the perpetuation of generations [25], which is realized through reversible polyploidy (ploidy cycles) that gave evolutionary origin to meiosis and sex [26]. This cancer unicellular life-cycle hypothesis was inspired by the observations of tumour giant cells in their prolonged response to ionising irradiation, finding the phenotypes comparable with those of some protists undergoing cycling polyploidy in their life-cycle [25,27,28,29].

From that standpoint, “dormant” polyploid giant cancer cells (PGCCs), which are heavily implicated in resistance, are presumed to represent a kind of “cancer germline” that is reciprocally linked to a mitotically dividing cell line, cyclically renewing its immortality [30].

The reproductive role of cancer polyploidy has been intensively studied and reviewed over the last two decades in our and about 20 other laboratories, and also earlier, becoming more and more recognised worldwide. It was shown that a single PGCC is able to induce a metastatic tumour at xenotransplantation [31] and appears similar to an early embryo [32,33,34,35,36,37,38,39,40,41,42]. A summary table of these studies is available in [43], the historical arrow diagram of cancer polyploidy research milestones—in Moein et al. [42], the latest advances united in a special issue [44]. This, along with the exchangeability of tumour and embryo nuclei and cells, already shown in the 20th century, has thus awakened and led to the emergence of a new, polyploidy-related twist on the embryonal theory of cancer, which initially originated in the 19th century [32,33,34,35,36,37,38,39,40,41,42].

Meiosis requires a tetranemic bivalent from two replicated parental homologous chromosomes for the main event of meiosis I—meiotic recombination and crossover, which effectively repair DNA, prevent accumulation of deleterious mutations with loss of heterozygosity (LOH), and provide genetic diversity [45,46]. The next important role of meiosis is the ordered halving of DNA content (suppressing one DNA replication cycle between two cell divisions), which might be involved, as suggested in [11,47], in the depolyploidisation of PGCCs whose progeny can start a new life-cycle.

In multicellular organisms, the germ cells are either specified during embryonic development or generated from somatic cells in a soma-to-germ transition (e.g., in plants, this is a very common phenomenon) [48]. Bruggeman et al. [14], who had observed the widespread expression of germ cell-specific (from embryonic to adult) genes in a wide database set from cancer cell lines and primary malignant tumours, proposed the involvement of such a soma-to-germ transition process in cancer evolution; the same was proposed by [5,8,34], while we have reported the induction of the embryonic stem cell-like markers (POU5F1, SOX2, NANOG) starting after DNA damage together with the polyploidy cycles [49]. In turn, cancer–testis (CT) genes are also considered cancer stem cell biomarkers [50,51]. The expression of cancer–testis genes may contribute to the ploidy cycle in both its poly- and depolyploidisation stages. Some of them are involved in meiosis and germ cell development, and some, particularly members of the Melanoma Antigen Gene (MAGE) family and PRAME, are implicated in the downregulation of the *p53* tumour suppressor [52,53,54], epigenetic reprogramming to stemness, and germline initiation [55,56], along with suppression of differentiation [57] and poor clinical prognosis [56,58].

However, it still remains to be seen whether this association between GG expression and cancer is pre-programmed (correlated, networked, and module-hubbed), or, as suggested by some authors [59], coincidental and random. On the other side, between those who do not doubt the programmatic atavistic development of cancer, there is a controversy over the time of its evolutionary origin, unicellular or early multicellular [60,61,62]. More fundamentally, there is also no clarity on the functional role of the meiotic toolkit in polyploidy-including life-cycles of the obligatory agamic protists [63,64]. In this study, we approach these questions by means of complex network analysis on publicly available cancer patient datasets (transcriptomes, inferred ploidy/WGD values, phylostratigraphy, and proteomes). To explain our goals, it is important to highlight the prerequisites in more detail.

Firstly, Stuart Kauffman, and then together with Sui Huang and Ernberg, proposed an idea that human cancer is driven by an attractor of the genome network that was evolutionary pre-formed (but not used) near the top of the onto-phylogenetic tree [65,66]. Indeed, phylostratigraphic studies revealed the evolutionary origin of cancer-driving genes [67] and a cancer transcriptome evolutionary gene profile shift to the emergence of multicellularity from unicellularity [60,68,69,70], likely disrupting the normal gene balance between uni-and multicellularity gene expression in the mammalian genome [61]. Interestingly, the earliest naturally occurring tumour was observed as far back as in the basal Eumetazoan *Hydra* [71]. However, the role of polyploidy in this phylostratigraphic shift of primary tumours, to our knowledge, has not been investigated.

Polyploidy as such (in normal mammalian tissues) induces a similar epigenetic shift favouring upregulation of unicellular and early multicellular genes (phylogenetic strata 1–5, from Prokaryotes to Eumetazoa), accompanied by downregulation of the genes of complex multicellularity, responsible for apoptosis, differentiation, immunity, and cell communication [72]. This shift towards unicellularity is aided by the ploidy-upregulated key proto-oncogene *c-myc* (originated in Opisthokonta) opening the chromatin for reprogramming [73], leading to epithelial-mesenchymal transition (EMT) [72,74,75,76] and hubbing the network nest of upregulated bivalent genes (in the same phylogenetic strata 1–5). The latter enable a critically rapid switch of the gene promoter activity from a suppressed to an active state. Moreover, cancer gene ontology (GO) modules embracing the c-MYC-HRAS axis and EGFR nest become activated by polyploidy [72,74,75,76].

Here it is worth adding that the current experimental evidence of cancer’s resistant response to conventional therapy can be summed up in three stages: induction of cellular senescence (with telomere attrition and persistent DNA damage signalling), polyploidisation, and reciprocal reprogramming/self-renewal [77]. The link between stress-induced accelerated cell senescence and reprogramming has been shown in many studies [30,78,79,80,81].

The meiotic pachytene recombination checkpoint has initially evolved from the mitotic G2/DNA damage checkpoint and uses some proteins from it [82,83]. As such, the persistent DNA damage induced in accelerated cellular senescence by oncogenes, drugs, or oxidative stress may enable cancer cells to slip from the mitotic DNA damage checkpoint into the meiotic prophase, as suggested in [77], assuming a polyploidisation variant in the form of Mos-driven endomitosis [27]. The other (or the same) route are the cycles of so-called mitotic slippage (reversal of metaphase arrest after DNA re-replication, not accomplishing mitosis) expressing the meiotic proteins (DMC1, SPO11, MOS, REC8, STAG3, SCYP1, SCYP3, POU5F1) observed in vitro on the irradiated *p53*-mutant lymphoma cell lines [12,27], and doxorubicin-treated basal breast cancer cell line [81] and some meiotic genes in luminal MCF7 cancer after TOPO I inhibitor [84].

**Figure 1:**
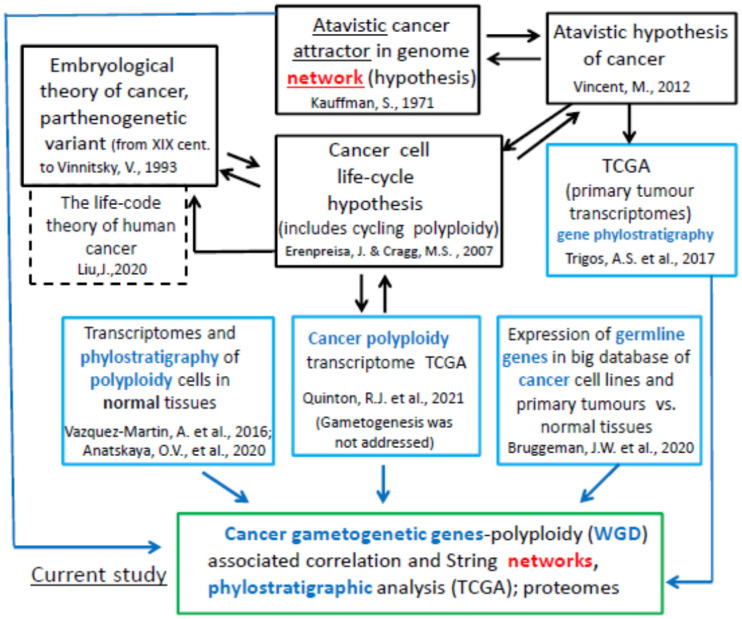
The context, aims, and design of the current bioinformatics study: in black boxes, the theoretical prerequisites; in blue boxes, what has been performed in previous similar studies (see the citations in the reference list [14,25,38,41,60,65,69,72,74,86]); in a green box, the aims and design addressed in the current study.

The problem is that, although GG genes were found abundantly expressed in tumours and associated with poor survival of patients, thus assigning fitness advantage to cancer cells, the conventional meiosis has never been microscopically observed, but instead, the polyploidy associated with meiotic markers was found [30,85]. On the other hand, there exist valid gene phylostratigraphic results demonstrating the converging of both WGD and the origin of cancer-driving genes to the same paleontological period of early eukaryotic evolution (as indicated above). Moreover, Quinton et al. [86] detected differences between transcriptomes of diploid (WGD-) and polyploid (WGD+) in the Cancer Genome Atlas (TCGA) tumours highlighting suppression of immunity, but the relationship between polyploidy and meiosis was not addressed there. The evidence of GG expression in cancer might have a programmatic evolutionary significance, if not only their presence but also networking and cooperation were to be proven in the context of polyploidy. The theoretical basis of these insights from the literature and the bioinformatics work in similar studies converging to the aim and design of the current work is schematised in Figure 1. The goal was to evaluate, through bioinformatics analysis of GG genes and proteins, their programmatically atavistic link with cancer polyploidy and to define the relevant attractor(s) in the human genome.

In synthesis, our results rule out the purely coincidental/random hypothesis of GG expression in favour of the re-activation of highly structured latent coexpression modules. Even if in this work, we do not make any direct PGCC observation, the emerging picture of robust GG upregulation by polyploidy (WGD) in multiple tumour types, as well as the highly organized coexpression networks that include them, give a relevant (albeit implicit) support to this hypothesis. Moreover, the obtained results suggest the role of polyploidy in soma–germ transition by visiting and interconnecting cancer attractors, pre-formed in the human genome network, during the origin and development of reproductive life-cycles, along the whole Evolution of Life.

## 2. Results

### 2.1. Whole-Genome Duplication Enriches GG Genes in TCGA Cancer Transcriptomes

Upon filtering the WGD-related differentially expressed (DE) genes (from [86]) for GG genes (Table S1), we observed non-zero GG gene upregulation in 23 of the overall 29 tumour types. A total of 10 tumour types (BLCA, BRCA, GBM, LIHC, LUAD, LUSC, OV, PRAD, SARC, STAD) possess a statistically significant enrichment of GG genes among upregulated genes, compared to the whole transcriptome (right-tailed binomial test *p* < 0.05) (Table 1).

Of 29 cancer types, 7 (BLCA, BRCA, GBM, LIHC, LUAD, PRAD, SARC) used in the tumour WGD study by Quinton et al. [86] have >100 ploidy-upregulated genes, of which more than 10% belong to the GG group (the maximum being BRCA at 25.27%). Furthermore, the GG genes are clearly depleted among ploidy-down-regulated genes (left-tailed binomial test *p* < 0.05 in all cancer types with any down-regulated GG), with the maximum proportion of GG genes among down-regulated genes being just 3.54% (Table 1). A statistically significant (binomial test *p* < 0.05) trend towards GG upregulation rather than downregulation can be observed in 13 cancer types (BLCA, BRCA, GBM, HNSC, KICH, LIHC, LUAD, OV, PRAD, SARC, STAD, TCGT, UCEC). Furthermore, 17 of 29 tumour types have at least one gene from the GG group fall into the top-25 genes when ranked by the highest logFC. A total of 10 of these tumour cohorts have MAGE group members in their top-25 upregulated genes; 3 (BRCA, HNSC, LUAD) have both MAGEs and PRAME in their top-25 upregulated genes.

In an attempt to uncover the evolutionary meaning of GG gene upregulation in polyploid cancer, we next decided to investigate the evolutionary history of GG gene origin using gene-phylostratigraphic information.

### 2.2. The Phylostratigraphic Distribution of GG Genes

**Figure 2:**
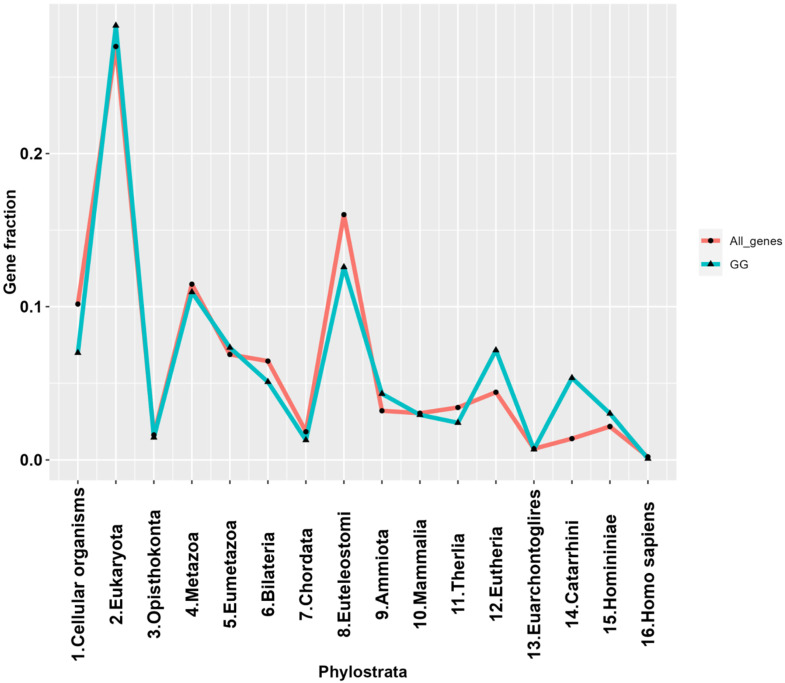
The phylostratigraphic distribution line plot of genes in the GG list (n = 1474), with all available genes serving as a reference.

After plotting the phylostratigraphic distribution of GG genes based on the gene phylostrata classification used by Trigos et al. [60], we observed significant peaks in phylostrata 2 (Eukaryota) and 8 (Euteleostomi) (Figure 2). Overall, this distribution was approximately concurrent with the reference of all available genes for which phylostratigraphy information was available. However, compared to the reference, GG genes show higher enrichment in the 12th and 14th phylostrata of placental animals and ancestral primates. The list of GG genes per phylostratum is presented in Table S1.

**Figure 3:**
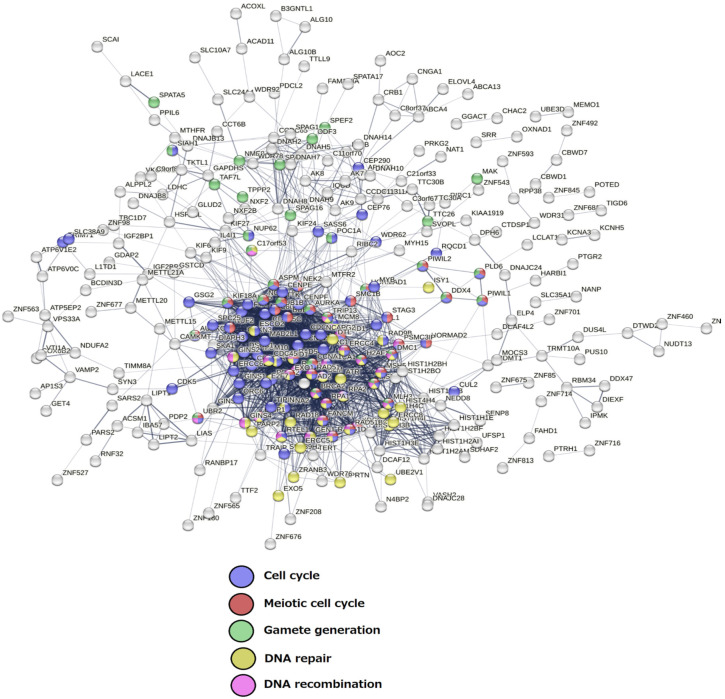
The STRING network of GG genes in the 1st and 2nd evolutionary phylostrata with enriched GO biological process modules highlighted in colours.

Briefly, from this GG list, it can be seen that the powerful meiosis-specific recombinase *DMC1* (the homolog of bacterial RecA which appeared in the Archaea in two forms [87,88]), along with the recombinase *RAD51*, fall into the Prokaryotic stratum 1, while most of the meiotic recombination toolkit with *HORMAD* (which can organise the axial element for chromosome pairing [89], and the meiotic Aurora kinase variant C (cooperating with mitotic AURKB), are already present in the 2nd Eukaryota stratum. DNA damage response elements (*ATR* and *CHEK1*, integrating ATR and ATM signalling) also appear in strata 2 and 3, respectively, and the *MOS*-kinase, responsible for the main steps of female meiosis—the recombination-dependent monopolar spindle and oocyte maturation [34,90,91]—in stratum 4 (Metazoa). Stratum 5 adds *PRDM14*, *PRDM9* and *DAZL* for the commitment of primordial germ cells. The important element of the conventional meiosis—the protein of the synaptonemal complex central element (SYCP1), together with the SGO stabilisers of the meiotic centromeric cohesin REC8, developed by stratum 8, coinciding with the Cambrian explosion. Late strata 12 and 14, starting from the Eutherians (placental mammals), added the majorly X-chromosome-linked CT genes (*PRAME*, most *MAGEA* genes in stratum 12, the *GAGE* and *PAGE* groups in stratum 14 (Catarrhini—Old World monkeys)—normally found in both gonads and the placenta [92]. The *Homo sapiens* stratum 16 added only the *STAG2* component of the meiotic cohesin complex. The STRING network representation of the GG genes falling into the respectively oldest and dominant 1st and 2nd strata can be seen in Figure 3.

**Figure 4:**
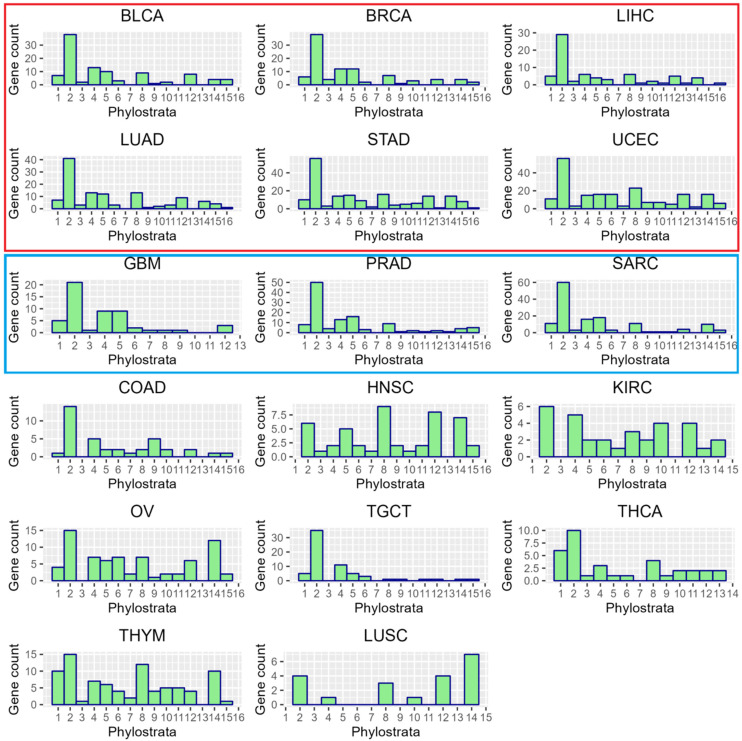
The phylostratigraphic distribution of ploidy-upregulated GG genes in 17 primary TCGA tumour types (that meet the criteria of having >10 ploidy-upregulated GG genes). The histograms of the 6 tumour types whose STRING and coexpression networks of upregulated genes are enriched for meiosis and gametogenesis-related GO and KEGG modules are highlighted in red, the 3 tumour types for which the former is true only in the case of STRING networks—in blue. Notably, they have a similar pattern of phylostratigraphic distribution of GG genes along the evolutionary tree, which is also similar in COAD and represents the most frequent types of somatic human cancers. Abbreviations are the same as in Table 1.

It is seen that the meiotic cell cycle appeared in Eukaryota, coupled with the recombination DNA repair, along with sex (gamete generation). Applying the same method to ploidy-upregulated GG genes in the TCGA dataset (Figure 4) revealed a dominant peak at phylostratum 2 in 15 of the 17 tumour types eligible for analysis (having >10 upregulated GG genes), along with high variability of the other part of the histogram occupying the whole evolutionary timeline, still highlighting a notable peak at stratum 8 (save for the TGCT cancer, which is also depleted of the later ploidy-upregulated GG genes). In all other tumours including PRAD, CT genes of the strata 12–14 (placental and hominid animals, respectively) are more or less overexpressed; in LUSC, this peak is dominating. The CT genes found, besides tumours and testis, in normal ovaries and placenta, inspired Lloyd Old’s witty conclusion: “Cancer is a somatic cell pregnancy” [93]—a paradox which will be later discussed.

**Figure 5:**
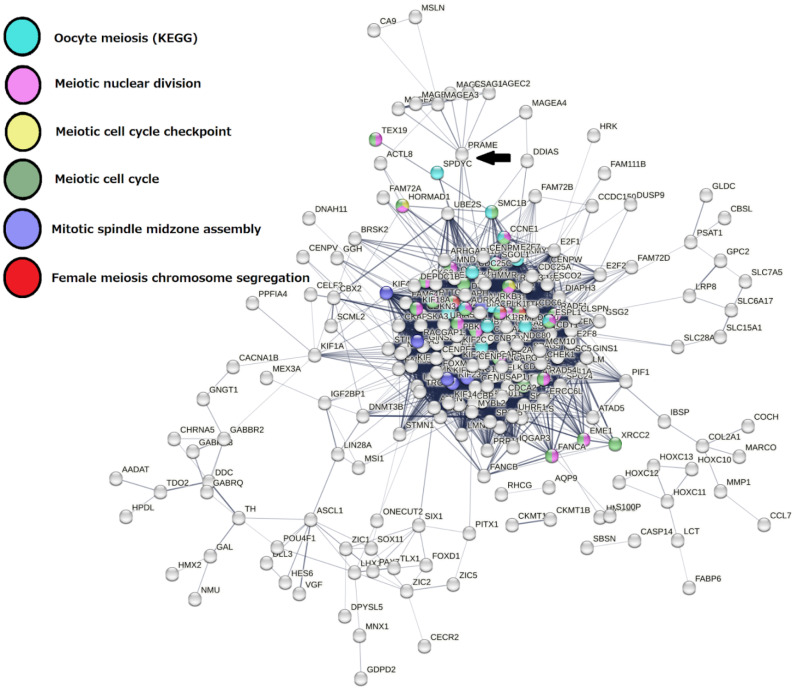
A STRING network of the ploidy-upregulated genes in TCGA-BRCA with colour-highlighted genes of meiosis-related enriched GO and KEGG modules (at medium confidence) connected by PRAME to a CT gene cluster (MAGEs) (arrow).

Examining the STRING protein–protein interaction (PPI) networks of the 29 TCGA tumours at both medium and high confidence revealed that nine of them (bladder carcinoma, breast carcinoma, glioblastoma, liver hepatocellular carcinoma, lung adenocarcinoma, stomach adenocarcinoma, prostate adenocarcinoma, sarcoma, uterine corpus carcinoma) possess the ploidy-upregulated meiotic GO and KEGG modules. An example of such a network in BRCA samples is shown in Figure 5. It demonstrates a tightly associated sub-network of the meiotic cell cycle with strictly meiosis-specific (i.e., HORMAD1, MND1, SMC1B) genes and includes CT genes such as TTK and PBK (known also for metastatic prostate carcinoma [94]). Furthermore, the giant sub-network is connected through PRAME to a cluster of MAGE-family of CT-proteins, which are considered to be metastasis-favouring oncogenes playing an important role in the soma-to-germ transition—by epigenetic reprogramming, germ commitment, and differentiation suppression (reviewed in Introduction).

In Figure 5, besides the elements of the meiotic cell cycle composed of functional nodes: meiotic cell cycle checkpoint (responsible for meiotic DNA recombination) and female meiosis including oocyte maturation, the polyploidy-related network contains an element of mitotic karyokinesis—spindle midzone assembly, which will be later discussed. The enrichment of GO and KEGG modules related to oocyte meiosis is seen in the six STRING networks of nine meiotic module-enriched tumour cohorts (Files S1).

Moreover, performing coexpression network analysis on six of the total nine meiosis module-enriched tumour types (the other three were rejected from coexpression network analysis due to not meeting both criteria of at least 100 upregulated genes and at least 50 polyploid samples with a purity > 0.5), in line with the STRING results, also revealed that the giant component of the ploidy-upregulated gene coexpression network is enriched for meiosis-related GO and KEGG modules in general, and modules relating to oogenesis/female meiosis in particular. The tumour types selected for coexpression network analysis are listed in Table 2.

**Figure 6:**
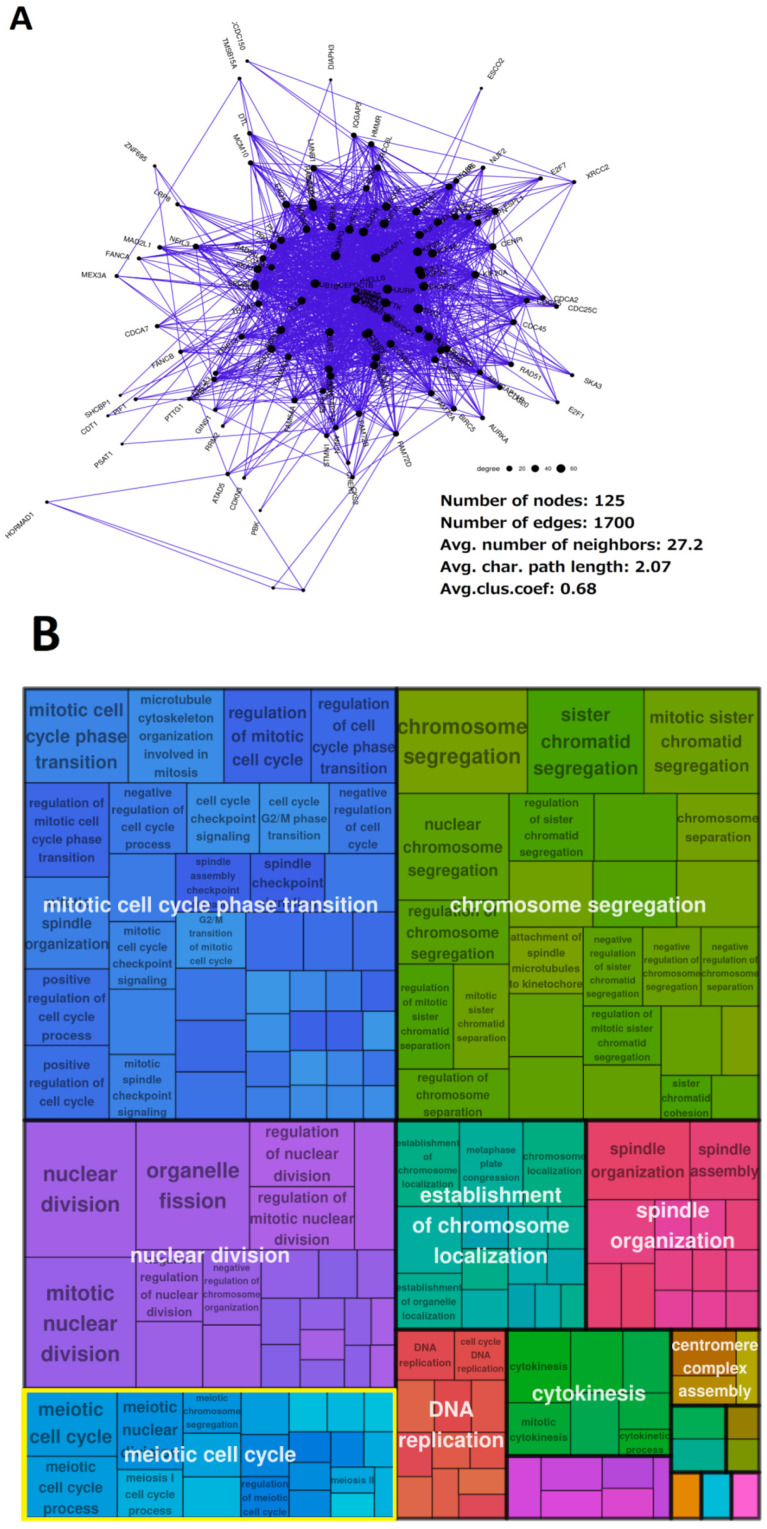
The cooperation of the ploidy-upregulated genes in TCGA-BRCA: (**A**) The giant component of the coexpression network of ploidy-upregulated genes. (**B**) A tree map plot of GO biological process modules enriched in this network. Modules related to meiosis are highlighted (yellow).

The analysis revealed a high proportion of the ploidy-upregulated genes associating into a network enriched with the meiotic and female-specific meiotic GO/KEGG modules with high average clustering coefficients. An example of one such coexpression network (TCGA-BRCA), with 125 nodes, 1700 edges, and an average clustering coefficient of 0.68 can be observed in Figure 6A, and the functional enrichment results thereof in Figure 6B. The rest of the networks have been deposited and are publicly available in the Network Data Exchange (NDEX) database (see Data Availability Statement below).

#### The Senescence Module of the Ploidy-Upregulated Gene Network

Among the six tumour types with highly WGD-upregulated GG genes and enriched meiotic (among them, female-specific) modules in both STRING and coexpression networks, three (BRCA, LUAD and LIHC) demonstrated the enrichment of the cellular senescence KEGG module (seen in File S2). There, we found that the well-known G1/S transition inhibitor CDKN2A (encoding p14 and p16) and the upregulation of CHEK1 Checkpoint kinase 1 integrating signals from ATM and ATR, the two cell cycle proteins involved in DNA damage responses G2M transition regulation, also associate with chromatin in meiotic prophase. Additionally, FOXM1 participating in DNA damage response should be highlighted. The upregulated member of the MYB family of transcription factor genes family MYBL2 has been shown to activate cyclin D1 (involved in polyploidisation) and interact with multiple insulin-like growth factor-binding proteins [95]. This auto-regulating loop is associated with the frequent deregulation of the insulin growth factor signalling pathway in cancer [96]. Interestingly, 14 of the 29 TCGA cancer types, including the three with the enriched senescence module, show ploidy-upregulation of IGFBPs and IGF2BPs, IGF2BP1 and IGF2BP3 being the most frequently encountered. IGF2BP1 and IGF2BP3 are oncofoetal proteins that are involved in stem cell renewal, organogenesis, and gametogenesis [97,98,99], and are also listed as MYBL2 interactants in the Harmonizome database [95].

This may be related to the fact that senescence induces DNA double-stranded breaks (DSBs), which, viewed in the context of GG gene expression and meiosis, may be interpreted as conferring the capability for a soma–germ transition from the mitotic DNA damage checkpoint in G2 (equivalent to WGD, in case of interrupted mitotic cell division, as discussed in Introduction). The insulin-like growth factor (IGF1)-related pathways may play the same or a converging role in a soma–germ transition. Insulin can substitute progesterone for inducing female oogenesis through direct activation of MOS by Ras upregulation in senescent somatic cells [34]. IGF1-related pathways are activated by hypoxia/an acidic environment [100].

As for the ploidy-down-regulated genes, the results of GO and KEGG enrichment analysis on both STRING and coexpression networks largely skew towards immunity-related processes in all nine analysed GG-enriched tumour types. The same was shown by [74] as related to polyploidy in normal tissues, and by [86] as related to polyploidy in tumours. Modules related to apoptosis and tissue homeostasis are also stably present in the ploidy-down-regulated gene networks (File S3).

### 2.3. Malignant Melanoma (MM) and BRCA Proteome Analysis Reveal Robust Expression and Coexpression of GG Proteins

In order to investigate GG gene expression and coexpression in cancer on the protein level, we selected and analysed two recently published high-throughput proteomics datasets of MM and BRCA.

#### 2.3.1. The Malignant Melanoma Proteome

Hierarchical clustering had stratified the MM500 database [101] patient cohort into six clusters (Figure S1). Taking into account the limited available clinical data, as well as the presence of non-random missing patterns provoking the impossibility to discriminate the existence of distinct biological subtypes from a batch effect, we decided to focus on the largest distinctive patient subset (n = 142), or Cluster 3 on Figure S1 for further analysis.

Overall, in the whole MM500 database melanoma proteome matrix, 411 proteoforms of reproduction-related genes (382 unique gene IDs) were found to be expressed in at least 20% of the samples (101 of 505). Patient Cluster 3, which was selected separately for further analysis, demonstrated the expression of 513 such proteoforms (501 unique gene IDs) in at least 20% of its constituent samples (>28 of 142).

After calculating correlations, thresholding the protein pairwise correlation matrix at |0.6|, and transforming it into a binary adjacency matrix, it was revealed that the vast majority (n = 452) of the expressed GG genes are also significantly coexpressed, forming the giant component of a network with a total of 3035 edges. Upon analysing the network with base Cytoscape [102] functionalities, it was determined to have an average clustering coefficient of 0.34 and an average shortest path length of 3.43.

**Figure 7:**
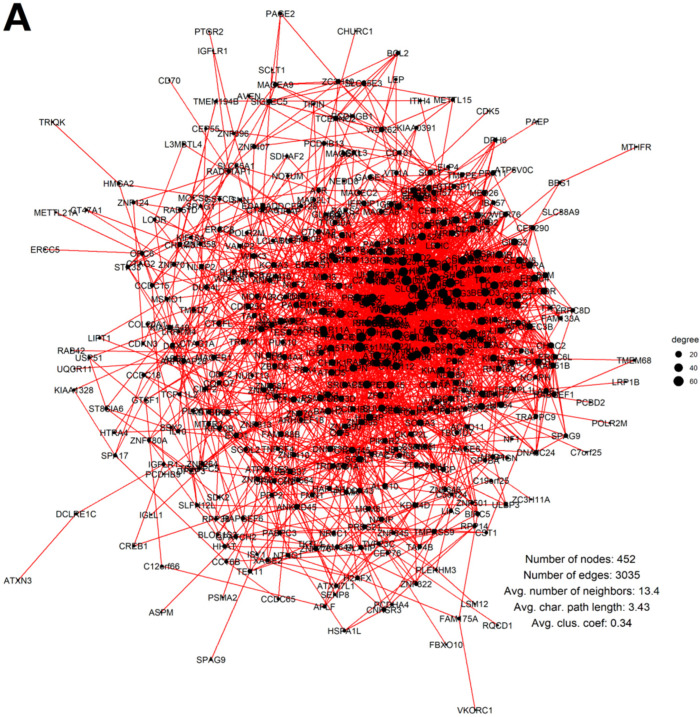

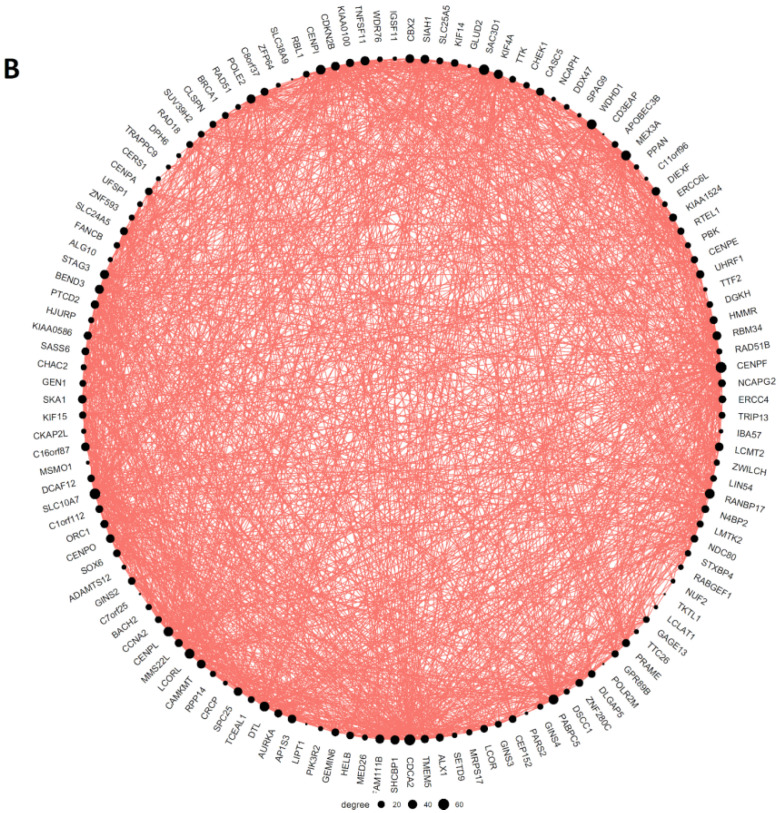
The coexpression networks of GG proteins in the MM500 database [101] melanoma proteomes (node size scaled to a degree): (**A**) The full network of GG proteins in 142 late-stage MM samples; (**B**) The most interconnected component (MCL Cluster 1) of the GG protein coexpression network, displayed in a circular layout.

Ranking the nodes in the network by degree (Figure 7A) reveals a highly interconnected “core” sub-network in its middle. Markov Cluster Algorithm (MCL) clustering at a granularity parameter of 2.5 extracted that interconnected component of 134 nodes and 1432 edges, with an average clustering coefficient of 0.58 and average shortest path length of 2.26 (Figure 7B).

In addition, GO biological process (BP) and KEGG enrichment analysis (right-sided hypergeometric test using all proteins identified in the MM500 proteome as the background, with the Bonferroni Step-Down (Holm) *p*-value adjustment method) identified meiotic GO and KEGG modules enriched in both the whole giant component of the network and, in particular, its most interconnected MCL cluster, including meiosis I, reciprocal meiotic recombination, female meiotic nuclear division, meiotic nuclear division, meiotic chromosome segregation, meiotic cell cycle checkpoint signalling, and blastocyst growth (Figure S2A).

#### 2.3.2. The Breast Carcinoma Proteome

**Figure 8:**
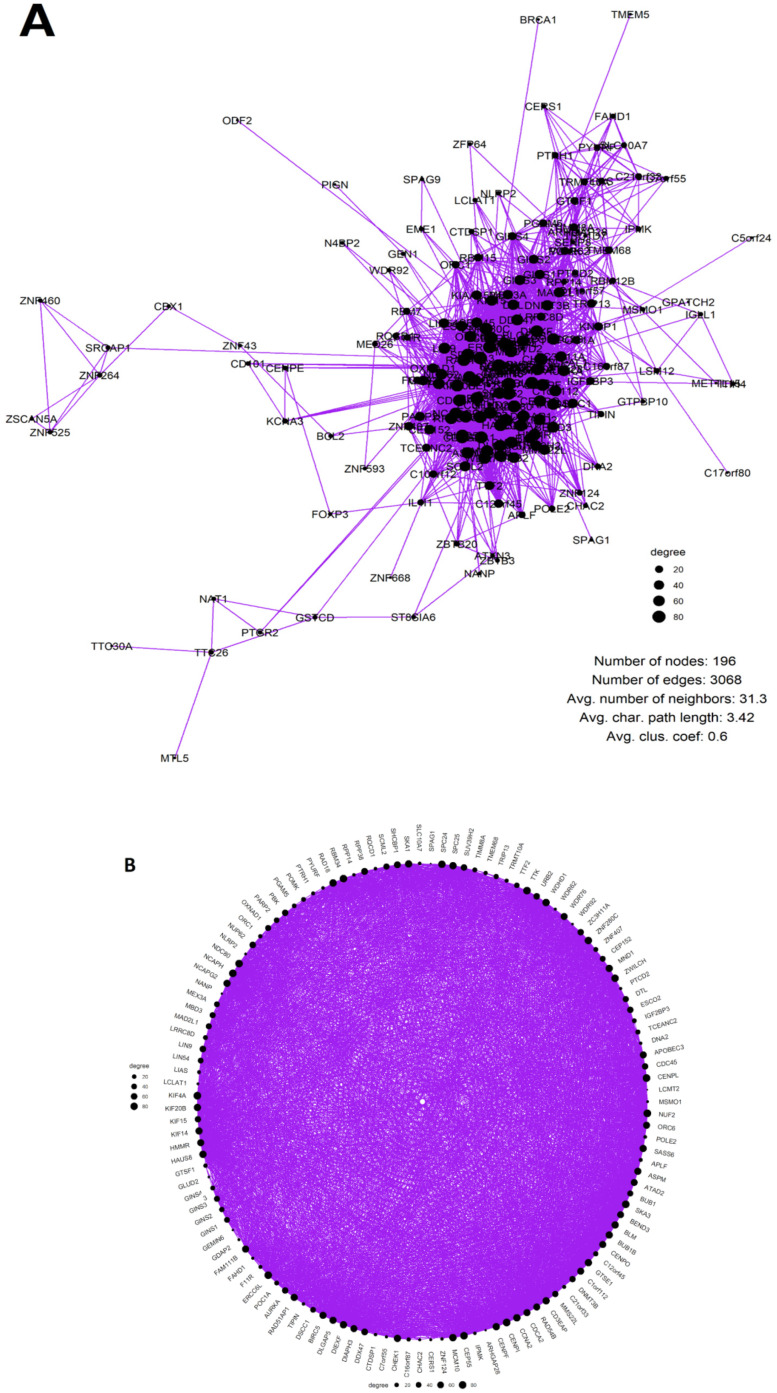
The coexpression networks of GG proteins in the PXD008841 [103] dataset of BRCA proteomes (node size scaled to a degree): (**A**) The full network of GG proteins in 45 BRCA tumour samples (**B**) The most interconnected component (MCL Cluster 1) of the GG protein coexpression network, displayed in a circular layout.

Overall, in the PXD008841 [103] proteome matrix of 45 BRCA (pre-filtered for insufficiently expressed proteins), 316 proteoforms of reproduction-related genes (316 unique gene IDs) were found to be expressed. A total of 196 (62%) of them were found to make up the giant component of a highly interconnected coexpression network (Figure 8A), with 3068 edges and an average clustering coefficient of 0.6. MCL clustering at a granularity parameter of 2.5 extracted the most interconnected component of 127 nodes and 2812 edges, with an average clustering coefficient of 0.77 and an average shortest path length of 1.86 (Figure 8B).

GO BP and KEGG enrichment analysis) identified meiotic GO and KEGG modules enriched in both the whole giant component of the network and, in particular, its most-interconnected MCL cluster (Figure S2B) including meiosis I, meiotic nuclear division, meiotic chromosome segregation and inner cell mass proliferation (Figure S2B). In this analysis, the right-sided hypergeometric test, using all proteins identified in the BRCA proteome as the background with the Bonferroni Step-Down (Holm) *p*-value adjustment method, was applied.

## 3. Discussion

In this study, we have addressed a working hypothesis that the genes involved in gametogenesis also cooperate in cancer development as part of a polyploidy-associated, coordinated, and pre-programmed process. To test the hypothesis and attempt to “capture” evidence of this cooperation and ploidy association, we have performed a bioinformatics study, including network analysis, on twenty-nine transcriptomic and two proteomic datasets of malignant tumour patient samples.

In this context, it is important to mention the limitations of the methods employed to acquire the results. This study focuses on bulk-sequenced samples, which complicates the capability to fully assess the complexity of the heterogeneous tumour. As such, the limitations of bulk transcriptome sequencing restrict the analysis of the biology of PGCCs, which are hypothesized to be the reproductive component of cancer, but may represent a very small part of the population, increasing in numbers when the tumour undergoes oncogenic and oxidative stress displayed as reversible cellular senescence [77]. The analysis to identify differentially expressed genes in bulk RNA-seq samples also only assesses the difference between WGD+ and WGD− samples, while taking into consideration sample purity (the proportion of cancerous cells), but not the cancer cell population heterogeneity. We partially bypassed the above-mentioned limitations with the phylostratigraphic analysis of GG genes in WGD+ tumours.

With these restrictions and rigorous statistical selection criteria for each part of our bioinformatics analysis, we obtained “an upturned pyramid”: from 17 of 29 tumour types with >10 ploidy-upregulated GG genes, 9 have ploidy-upregulated gene STRING networks enriched with meiotic GO and KEGG modules, and 6 of these 9 tumour types that met the criteria for coexpression network analysis also showed coexpression networks enriched for meiotic modules in general and female meiotic modules in particular, while 3 of the latter (BRCA, LUAD, LIHC) also displayed the KEGG module of cellular senescence (which likely increased the proportion of PGCCs in them).

Altogether, despite the aforementioned limitations, the results obtained in this study show that, in a considerable number of tumour types, gametogenesis-related genes are not only robustly expressed (by the hundreds), but also coexpressed on both the transcriptome and proteome levels and associated with whole genome duplications. As previously mentioned, PGCCs (induced in various tumour cell lines after severe anti-cancer treatments and also found in smaller quantities in the control samples), were shown to be positive for meiotic proteins (MOS, REC8, DMC1, SPO11, POU5F1, DDX4, IFITM3), as revealed in individual cells by immunofluorescence [13,27,81,104,105] and time-series qPCR analysis after genotoxic treatments, along with senescence markers [7,8,9,10,11,12,13]; [13,27,81,104,105]. It is therefore logical to consider that these findings complement the evidence of a link between polyploidy and GG genes from the above-mentioned polyploid cancer single-cell immunofluorescence studies and reinforce the hypothesis. Interestingly, we have also observed that the networks of ploidy-upregulated genes in the datasets are also enriched for functional modules related to female meiosis (apparently regardless of patient sex), which thus favour cancer cell survival.

So, the evidence we currently possess—that of a robust GG gene (germ–meiosis–CT) network expressed in malignant tumours on both the transcriptome and proteome levels and showing links to polyploidy, oocyte development, and preimplantation embryogenesis—is indicative of a non-random pre-programmed process related to the reproduction.

But what kind of reproductive process could it be? Quite clearly, the process is (1) asexual and (2) hardly employs canonical gametic meiosis, which in its full form has never been observed in cancers [30,59,85]. It appears from current data that this cancer-, meiosis- and embryonality-related story is linked to polyploidy through its atavistic, evolutionary roots. In particular, the marked dominance of the phylostratum 2 for upregulated GG genes in WGD+ tumours points towards the association of cancer polyploidy with the origins of the eukaryotic life-cycle. As we have seen from the examined phylostratigraphic distribution of 1474 gametogenic genes, the origin of meiosis associated with DNA damage and recombination repair (the hypothesis of meiosis origin proposed by [106] appearing together with the origin of sex (gamete generation) in the very early eukaryote cells was confirmed here. In concord, another study on ancestral character state reconstruction for representatives of 106 eukaryotic taxa indicates that LECA (the last eukaryote common ancestor which likely gave rise to the first eukaryote cell (phylostratum 2) from a symbiosis of archaea and bacteria), in addition to possessing mitochondria, was sexual, meiotic, and multinucleate [107], and thus, polyploid. However, in the case of cancer, the reproduction process is an asexual one; it is thus worth mentioning some features of this kind of atavistic reproduction.

From the point of view of evolutionary genetics, the prevention of the so-called Muller’s ratchet [108]—the deleterious loss of heterozygosity (LOH) in asexual reproduction—can replace sexual reproduction (outweighing the “cost of sex”) only if it is associated with at least triploidy or tetraploidy [109], or if polyploidy is associated with inverted meiosis and/or cell fusion [110]. Archetti came to the conclusion that “polyploidy has a selective advantage against LOH shown for the evolution of different types of asexual reproduction in nature. This provides an adaptive explanation for cyclical ploidy, mitotic slippage, and cell fusion in cancer cells” [111]. As indicated by the author, DNA recombination in polyploids, occurring also between sister chromatids, can counteract LOH or act along with gene conversion. The latter mechanism was found in polyploid Archaea [112]. It follows that the mechanisms of genome protection by polyploidy were already in action in very early Eukaryota and, according to Kondrashov [26], gave origin to meiosis, which was immediately followed by sex. This mechanism possibly determines the earliest GG-related cancer attractor in the 2nd phylostratum, seen by us in all meiotic module-enriched TCGA WGD+ tumour types.

Indeed, asexual Amebozoa display cyclical polyploidy along with expression of the basic meiotic toolkit of 12–15 genes [63,113]. These genes have been observed in human tumours as well [14,81,114,115], while ameboid multi-nucleated cell patterns and even macrocysts were encountered in cancers treated with genotoxic agents [29,62,81,116]. The dominant basic 2nd phylostratum peak of meiotic recombination and DNA repair was in fact WGD-upregulated in 15 of the 17 eligible tumour types. It is indispensable for any sexual or asexual reproductive process, as it processes the function of meiosis I, which may also be non-conventional, e.g., inverted [81,110] or preceded by biparental genome segregation [23,117]. The budding or bursting of offspring from PGCCs followed in some models after one or several multi-nuclear-bridged reconstruction cycles, preceding the cellularisation of subnuclei by postponed radial cytotomy. This kind of nuclear reconstruction (including pedogamic exchange of the division products in tripolar a-cytotomic karyotomy) of the giant cell shows further the extended central spindles associated with Aurora B kinase converging to a monopolar composed centrosome [118,119]. This process was particularly typical for irradiated HeLa and lymphoblastoma, but also described in senescent mouse fibroblast cultures [119]. It can explain the presence of the mitotic spindle midzone assembly GO BP found enriched in the STRING network of WGD+ BRCA (Figure 5). Further, these cells underwent cellularisation of subnuclei and disintegration by bursting or budding. Such cell division with postponed cellularisation within multinuclear cells created by a-cytokinetic karyotomy is known in evolutionary biology terms as coenocytosis, which is often associated with reproduction [120]. It is very likely that similar reproductive adaptations could originate at the early transitions from uni- to multicellularity [121].

Besides the basic cancer attractor in the 2nd phylostratum, we observed in the TCGA tumour dataset of ploidy-upregulated GG genes the engagement of the phylostrata of early multicellular organisms (strata 4 and 5), where the oocyte meiosis and preimplantation embryo were already evolutionarily stabilized.

The origin of cancer driver genes is also focused on the first five phylostrata, embracing the origin of unicellular and embryonic reproduction life-cycles and supporting the atavistic ancestry of cancer [60,67,69,70,122].

Our GG phylostratigraphic study also revealed a large group of CT-genes in strata 12–14, for placental animals and Old World monkeys; some were evolutionary amplified in the hominid genome. These CT-GGs were also ploidy-upregulated in TCGA tumours. In particular, they include the MAGEA, B group, multiple SPANX members, and PRAME, which binds CT-GG cluster to the giant meiotic cluster of the human WGD+ cancer network. In fact, this is also a cancer attractor conferring stemness, germline induction, loss of differentiation, immunity modulation, metastases, and poor clinical prognosis [56,58]. It may further reinforce the embryonic attractor by insulin-like-growth factor-related, progesterone-independent pathway [30,34]. One of the CT protein families, SPANX, warrants special attention in association with the budding of survival descendents from PGCCs, claimed by many authors to be amitotic [123,124]. In this context, it is necessary to refer our electron microscopic study applied after 10Gy irradiation or mitotic spindle inhibitor SK&F in Burkitt’s lymphoma cell lines. Within induced multiple PGCCs, the intra-cytoplasmic sequestration of nuclear buds and micronuclei was revealed to be started by annulate lamellae (AL), the derivates of the nuclear envelope, branching from the nuclear membrane of the main nucleus, along with the emergence of the folds of the nuclear envelope limited chromatin sheets (ELCS). The process was occurring at the brink of mitotic death with survival of <1% PGCCs resistant cells producing offspring [125]. A somewhat similar transformation of the nuclear membrane with blistering of ELCS and AL is occurring in spermiogenesis, reducing the nuclear volume by blebbing the redundant nuclear envelope into the mammalian sperm cytoplasmic droplet [126]. This process is regulated by SPANX, which is a component of lamin A [127]. The Xq27-located CT-protein SPAN-X family is overexpressed in melanoma, testicular, breast, prostate, lung, and other cancers [126]. Transfection of SPANXA into mammalian cells causes nuclear budding and micronucleation, which are also characteristic of senescing giant cancer cells [127]. Interestingly, the SPANX family originated in rodents from SPANX-N, which is located in the acrosome for sperm motility, and split (via locus amplification) into the SPANX-A/D group specific for hominids (phylostratum 14) with a new function—that of nuclear membrane reduction in spermatids [128]. Some other X-chromosome-linked CT genes (mostly MAGEA group) also appeared late in evolution together with placentation and were amplified in hominids. This may be associated with the human trophoblast developing polyploid giant cells with high invasiveness, which could be evolutionarily necessitated by the large size of the primate foetus and the prolonged pregnancy period [129]. It is also interesting to note that the evolution of placental invasion and cancer metastasis appears to be causally linked [129,130], sharing as reported IGF/MAPK, BCL2, Wnt-signalling [131,132] and, most importantly, immune evasion.

## 4. Materials and Methods

### 4.1. TCGA Polyploid versus Diploid Tumour Transcriptome Comparison

We followed a strategy of analysis tailored upon Quinton et al. [86], who bioinformatically detected and experimentally validated differences between transcriptomes of diploid (WGD−) and polyploid (WGD+) TCGA tumours. The approximate ploidy of analysed samples determined by the ABSOLUTE algorithm (which uses whole-genome copy number information to reconstruct the trajectory of tumour genome evolution and estimate the presence of WGD) [133] was obtained from the supplementary data of Taylor et al. [134]. DE genes obtained from the supplementary materials of [86] were filtered by the adoption of the inferential/effect-size threshold: (pAdj < 0.05, |logFC| > 0.5).

In order to assess the possible presence of germ–soma transition and/or pseudo-meiotic features related to polyploidy in the TCGA datasets, a combined gametogenesis-related (GG) gene set comprised of cancer–testis genes from the CTDatabase [135], cancer–germ cell (primordial and adult male) genes from [14] and the MeiosisOnline [136] human meiosis-involved gene database that was updated with a manually-curated set of additional genes (SYCP1, SYCP2, SYCP3, SYCE1, SYCE2, HORMAD2, MAEL, MEIKIN, MEIOB, MEIOC, SYC E1L, TEX11, MAJIN, FAM9C, FAM9B, FAM9A, REC114, TEX19, BRME1, TEX14, MSH4, TEX15), numbering a total of 1474 genes, was used to filter the ploidy-upregulated and downregulated differentially expressed (DE) gene lists. To test for enrichment or depletion of GG genes among DE genes, binomial tests were performed with the R stats package. Information on gene phylostratigraphy was obtained from [60] and phylostratigraphic distributions of GG genes were visualised with ggplot2.

The potential relationship between ploidy and reproductive features was further investigated by constructing networks (a STRING [137] PPI network and a coexpression network). The STRING web interface was used for constructing the STRING networks from the filtered lists of DE genes. The coexpression network analysis was used on cancer types that were shown to have meiotic modules enriched in their STRING networks at least at medium confidence, in order to assess whether these genes are indeed cooperating in this particular dataset.

For the construction of coexpression networks, Rsubread-processed TPM-normalised TCGA data for 9264 tumour samples and 741 normal samples across 24 cancer types [138] were obtained from the Gene Expression Omnibus repository (GSE62944). Using the ABSOLUTE-calculated values, the data for each cancer type were split by the presence or absence of whole-genome duplications (into WGD+ and WGD-, or diploid and polyploid sample cohorts). To minimise the impact of tumour heterogeneity, a purity cutoff of 0.5 was implemented. Of the nine cancer types shown to have meiosis-related modules enriched in STRING, six of them met the conditions for at least 100 ploidy-upregulated genes, and at least 50 WGD+ samples post-purity cutoff (BRCA, LUAD, STAD, UCEC, LIHC, BLCA) were selected for coexpression network construction. The gene expression matrices were filtered for lowly expressed genes with a cutoff of at least 2 TPM in at least 20% of the samples.

The coexpression network (unsigned, with both positive and negative correlation coefficients counting as an edge) was obtained by computing pairwise Pearson correlations between DE genes.

A hard threshold of pairwise correlation coefficients was determined by comparing the number of links (pairwise correlations equal to or exceeding the threshold in absolute value) between 50 randomly picked sets of 300 genes and surrogate data—shuffling of these datasets across columns (50 shuffling for each set). This approach serves to determine a “mean correlation field” linking the genes (or, in the case of the proteomic data described in the next sections, the proteins), and the amount of noise/randomness present in the data [139]. In this case, a list of four possible thresholds, ranging from 0.6 to 0.9, were tested in this manner, and a threshold of 0.6 was found to be sufficient to define an edge in the network, with the number of interactions in “real” data vastly and highly significantly (Wilcoxon test *p* < 0.001) exceeding that of surrogate data (as seen in Table S2), indicating that the normalisation procedure in the initial data was successful at reducing the noise.

The selected threshold was used to transform the correlation matrix into a binary adjacency matrix, which was imported into Cytoscape via the RCy3 R package [140] and the aMatReader app [141]. The giant components of the upregulated and downregulated gene networks were then extracted for further enrichment analysis to determine.

Gene-set enrichment (Gene Ontology (GO) and Kyoto Encyclopaedia of Genes and Genomes (KEGG)) was adopted (using clusterProfileR in R and ClueGO in Cytoscape for visualisation of enrichment maps) as a proxy of genes’ biological functions, and the gene sets were checked for their statistical relevance by tests based upon hypergeometric distribution enrichment analysis with Bonferroni Step-Down (Holm) correction, which is more stringent in terms of false positives than the Benjamini–Hochberg correction implemented in the STRING web tool, for the *p*-value. For ease of interpretation, the large number of enriched processes was reduced, clustered, and visualised as treemap plots using the rvvgo R package [142] and/or ClueGO enrichment maps. Igraph [143] and graph [144] R packages were used to supplement Cytoscape’s network visualisation functionalities for interpretability and aesthetic reasons.

The NDEX database [145] was used for depositing the networks.

### 4.2. Analysis of GG Protein Expression in the MM and BRCA Proteomes

This analysis was performed to assess the relationships between GG gene expressions on the protein (whole-proteome) level. For that purpose, two high-throughput proteomics datasets of two cancer types (MM and BRCA) from public databases were used.

A matrix of normalised relative protein abundances determined by high-throughput LC-MS/MS from 505 late-stage melanoma patient tumour samples and over 12,000 protein-coding genes was obtained from the supplementary materials of the MM500 Melanoma Proteome Atlas study [101]. Hierarchical clustering (hclust in the R stats package) was performed to stratify the samples by abundance and value missingness. Cutting the tree at a height of 1400 (an ultrametrics based on Euclidean Distance) stratified the patient samples into six clusters (Figure 4). Patient Cluster 3 (n = 142) was selected for further analysis due to technical considerations.

The lowly expressed proteoforms were filtered out of the resulting 142-sample matrix, with a cut-off of at least five units of normalised relative expression in at least 20% of the samples. For the rest, the missing values were replaced using minimum imputation (the log2-scale value of the minimum possible measurement) with the assumption of low protein expression as the reason for their missingness.

A matrix of normalised relative protein abundances for 45 BRCA samples (mainly grades 2–3) was obtained from the ProteomeXChange PXD008841 repository [103]. Unlike the MM dataset, the BRCA dataset had already been filtered to only include proteins expressed in every sample, for a total of 9995 proteins. As such, no further low-expression filtering was necessary.

In order to assess the possible presence of soma–germ transition and/or pseudo-meiotic features related to embryonalization in late-stage melanoma and grade 2–3 breast carcinoma, the GG gene set (n = 1474) was used to filter out the proteins related to the aforementioned processes.

To investigate the relationship between the expressed proteins, the resulting GG protein abundance matrix was used to calculate pairwise correlations and construct a coexpression network with the same procedure as described in Section 4.1. In the proteome data, the threshold of 0.6 in absolute value was found to be sufficient, as can be seen in Tables S3 and S4. To determine the most interconnected network components or modules, the coexpression network was subjected to MCL clustering with a granularity parameter of 2.5, implemented in Cytoscape’s clusterMaker app [146]. GO Biological Process enrichment analysis was performed as described in Section 4.1, but unlike the case of the TCGA transcriptomes, in which all genes in the database were used, for proteomics data, the whole proteome of the cancer dataset in question (respectively, MM and BRCA) was used as the background gene set (“universe”). The enrichment analysis was done separately on the whole giant component of the protein–protein coexpression network, and its most highly interconnected part (MCL cluster 1).

The NDEX database [145] was used for depositing the networks.

## 5. Conclusions and Perspectives

We can conclude that human tumours can likely employ three or more cancer-polyploidy associated reproduction attractors of soma–germ transition, pre-formed and developed during life evolution on Earth. While the first, early eukaryotic and basic, is directly associated with DNA damage repair by recombination with the functions of the meiotic prophase, which seems indispensable for starting any reproductive life-cycle (and may be displayed in cancers by the amoeboid reproduction mode with an asexual macrocyst), the second, associated with oocyte maturation and early embryogenesis, is linked to the transition from unicellular to multicellular forms of life [60] (and can induce in cancers the parthenogenesis-like reproduction process). The current results provide a hint to the existence of a link between the WGD-related oocyte maturation and cellular senescence, as was suggested earlier [77]. The latest, placental attractor links the embryonic sub-network to germline determination in placental mammals through ectopic activation of PRAME/CT-genes (which presumably may favour “pseudo-placentation”—invasion and metastases). In addition, the coupling of the polyploidising mitotic slippage (that results in the acquisition of meiotic features) with the interruption of the circadian clock in WGD-positive TCGA cancers, shown by us recently [43], likely enables this scanning of the genome evolutionary memory through all aeons, revealing the permissive paths to cancer attractors of asexual reproduction and connecting the most adaptive among them into the very dense network disclosed in this study. Our data and their analysis confirm the view of the extreme adaptability of human cancers to the general pattern states of the genome network, neighbouring and distant tissues, and the microenvironment [147], by means of polyploidy-aided atavistic variable mechanisms of asexual reproduction. Tightly clustered and correlated gametogenesis- and WGD-related cancer networks found here in the common aggressive cancers present an argument in favour of epigenetic cancer evolution “as a model of cell learning”, providing its causal potential for anti-cancer therapies [148]. At the same time, carcinogenesis and tumour evolution remain very complex, and the present study is only a step to their better understanding.

## Figures and Tables

**Table 1:** The prevalence of GG genes found among ploidy-upregulated and downregulated differentially expressed genes (DEGs) *.

TCGA Cancer Type	% of WGD+ Samples	Number of Ploidy-Upregulated Genes	Upregulated Genes Enriched with GG (*p* < 0.05)	Number of Ploidy-Downregulated Genes	Downregulated Genes Enriched with GG (*p* < 0.05)
ACC	53.7%	84	FALSE	443	FALSE
BLCA	60.2%	424	TRUE	1000	FALSE
BRCA	44.0%	277	TRUE	1395	FALSE
CESC	33.7%	16	FALSE	157	FALSE
CHOL	22.2%	1	FALSE	0	FALSE
COAD	40.0%	431	FALSE	641	FALSE
ESCA	55.4%	0	FALSE	1	FALSE
GBM	17.6%	164	TRUE	850	FALSE
HNSC	39.4%	309	FALSE	867	FALSE
KICH	15.4%	164	FALSE	204	FALSE
KIRC	22.2%	305	FALSE	658	FALSE
KIRP	7.3%	36	FALSE	5	FALSE
LIHC	35.0%	435	TRUE	894	FALSE
LUAD	57.1%	431	TRUE	1445	FALSE
LUSC	54.5%	97	TRUE	1106	FALSE
MESO	22.6%	0	FALSE	4	FALSE
OV	57.6%	400	TRUE	1374	FALSE
PAAD	29.1%	11	FALSE	1083	FALSE
PCPG	21.4%	3	FALSE	6	FALSE
PRAD	11.6%	427	TRUE	610	FALSE
READ	52.3%	0	FALSE	4	FALSE
SARC	47.4%	573	TRUE	1040	FALSE
STAD	41.3%	1047	TRUE	928	FALSE
TGCT	95.3%	549	FALSE	523	FALSE
THCA	9.0%	686	FALSE	1138	FALSE
THYM	20.0%	1562	FALSE	1046	FALSE
UCEC	22.3%	1671	FALSE	1691	FALSE
UCS	82.8%	1	FALSE	1	FALSE
UVM	6.3%	13	FALSE	69	FALSE

Abbreviations: TCGA—The Cancer Genome Atlas, ACC—Adrenocortical Carcinoma, BLCA—Bladder Urothelial Carcinoma, BRCA—Breast Invasive Carcinoma, CESC—Cervical Squamous Cell Carcinoma and Endocervical Adenocarcinoma, CHOL—Cholangiocarcinoma, COAD—Colon Adenocarcinoma, ESCA—Esophageal Carcinoma, GBM—Glioblastoma Multiforme, HNSC—Head and Neck Squamous Cell Carcinoma, KICH—Kidney Chromophobe, KIRC—Kidney Renal Clear Cell Carcinoma, KIRP—Kidney Renal Papillary Cell Carcinoma, LIHC—Liver Hepatocellular Carcinoma, LUAD—Lung Adenocarcinoma, LUSC—Lung Squamous Cell Carcinoma, MESO—Mesothelioma, OV—Ovarian Serous Cystadenocarcinoma, PAAD—Pancreatic Adenocarcinoma, PCPG—Pheochromocytoma and Paraganglioma, PRAD—Prostate Adenocarcinoma, READ—Rectum Adenocarcinoma, SARC—Sarcoma, STAD—Stomach Adenocarcinoma, TCGT—Testicular Germ Cell Tumours, THCA—Thyroid carcinoma, THYM—Thymoma, UCEC—Uterine Corpus Endometrial Carcinoma, UCS—Uterine Carcinosarcoma, UVM—Uveal Melanoma. *—genes with >|0.5| logFC from the [86] dataset of 29 TCGA tumour type transcriptomes.

**Table 2:** Parameters of the ploidy-upregulated genes, the coexpression network thereof, and gametogenesis-related modules in (WGD+) samples of six TCGA tumour types *.

Tumour Type	Number of Samples (WGD+, >0.5 Purity)	Number of Upregulated Genes (pAdj < 0.05; logFC > 0.5) Expressed in High-Purity Samples	Percent of Upregulated Genes in the Giant Component of Network	Average Clustering Coefficient of Network	Meiotic GO/KEGG Modules Enriched in Coexpression Network (Y/N)	Female-Specific Meiotic GO/KEGG Modules Enriched in Coexpression Network (Y/N)
BRCA	277	220	56.8%	0.68	Y	Y
LUAD	103	340	52.9%	0.74	Y	Y
LIHC	91	290	94.5%	0.61	Y	Y
STAD	82	756	58.6%	0.47	Y	Y
BLCA	142	348	54.9%	0.5	Y	Y
UCEC	97	1217	48.5%	0.56	Y	Y

* These tumour samples were selected for analysis due to meeting the criteria of meiotic module enrichment in ploidy-upregulated gene STRING networks, at least 100 upregulated genes (as seen in Table 1), and at least 50 polyploid samples with a purity > 0.5. Y—Yes; N—No.

## Data Availability

The coexpression networks resulting from this study have been deposited in the NDEX database, with the transcriptome and proteome network sets available, respectively, under the following URLs: https://www.ndexbio.org/#/networkset/c4934da2-3cf7-11ed-b7d0-0ac135e8bacf?accesskey=5d269780b0440e6cf61633b03121b398598d19307be4c23760642ae33acbf749 (accessed on 5 October 2022). https://www.ndexbio.org/#/networkset/5e0ba954-3cf5-11ed-b7d0-0ac135e8bacf?accesskey=e29f7a1571fba879aa0b38158550baae147b7ea39a384f08f7c52a11a6165428 (accessed on 5 October 2022).

## Supplementary Materials

The supporting information can be downloaded at: https://www.mdpi.com/article/10.3390/ijms232314930/s1.
