# Supplementary figures and images for: The Transcriptome and Proteome Networks of Malignant Tumours Reveal Atavistic Attractors of Polyploidy-Related Asexual Reproduction
Source: Int J Mol Sci. 2022 Nov 29;23(23):14930. doi: 10.3390/ijms232314930 (PMC9736112; doi:10.3390/ijms232314930)

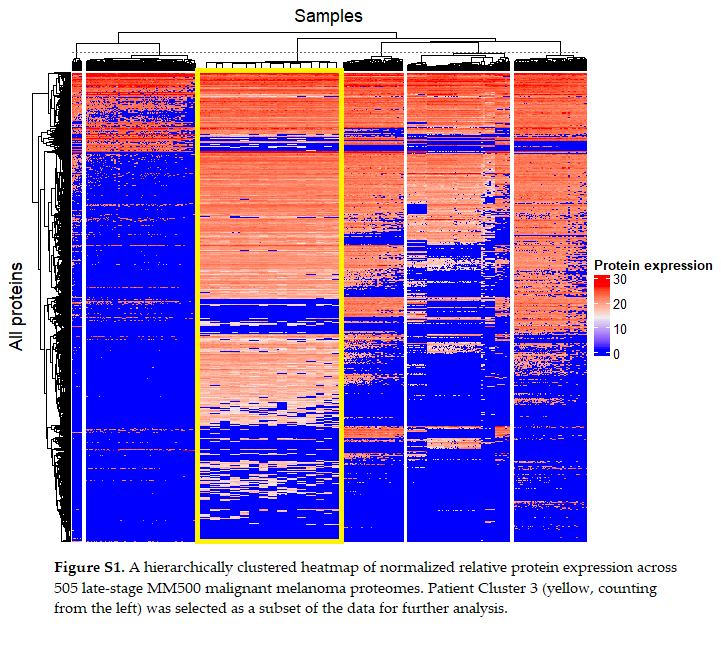

Supplement: Supplementary file 1 [file ijms-23-14930-s001.zip › Figure S1.tif]

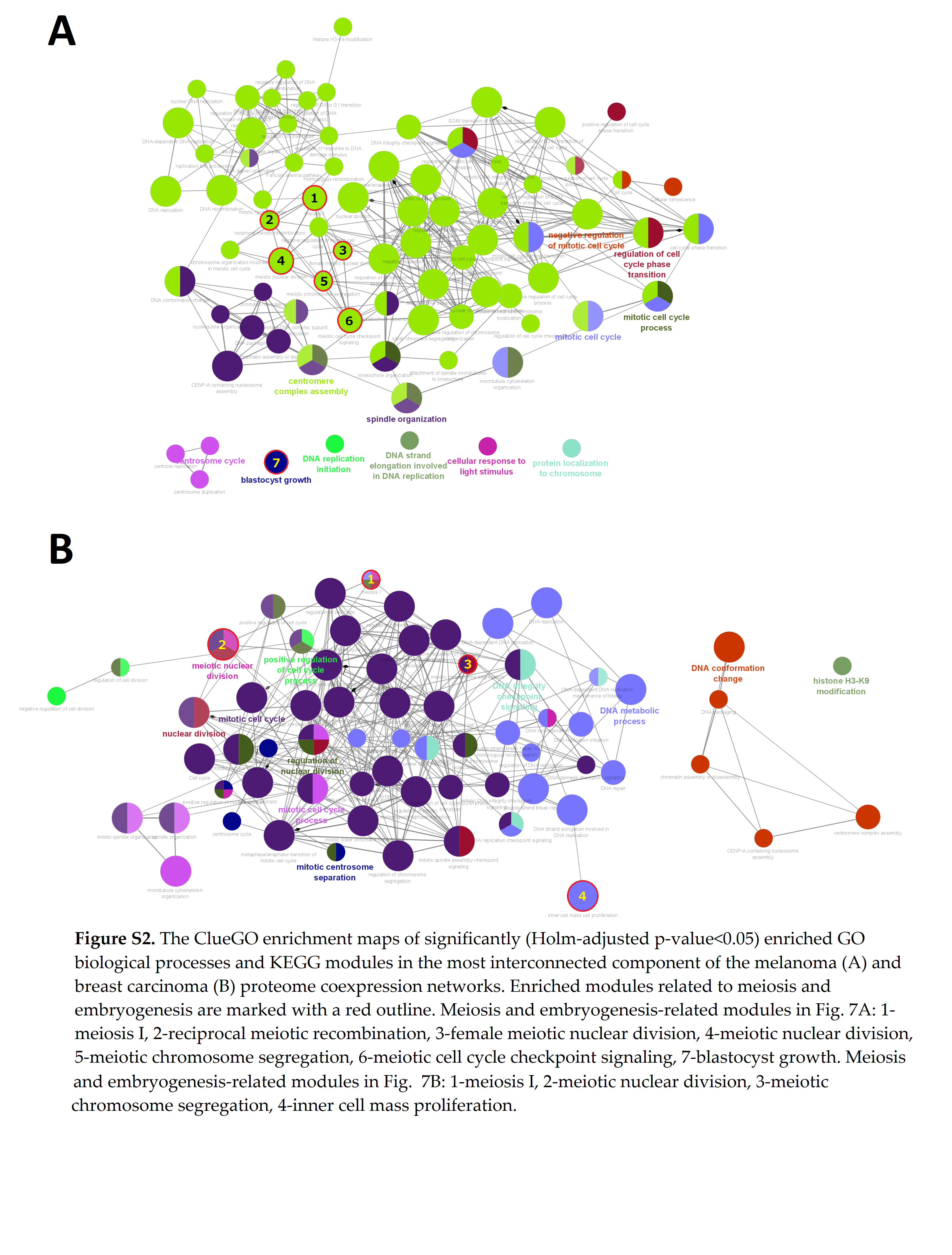

Supplement: Supplementary file 1 [file ijms-23-14930-s001.zip › Figure S2.tif]
